# Cytotoxicity, nitric oxide and acetylcholinesterase inhibitory activity of three limonoids isolated from *Trichilia welw*i*tschii* (Meliaceae)

**DOI:** 10.1186/s40659-015-0049-0

**Published:** 2015-10-13

**Authors:** Jean P. Dzoyem, Armelle T. Tsamo, Raduis Melong, Pierre Mkounga, Augustin E. Nkengfack, Lyndy J. McGaw, Jacobus N. Eloff

**Affiliations:** Department of Paraclinical Sciences, Faculty of Veterinary Science, University of Pretoria, Private Bag X04, Onderstepoort, Pretoria, 0110 South Africa; Department of Biochemistry, Faculty of Science, University of Dschang, P.O. Box 67, Dschang, Cameroon; Department of Organic Chemistry, Faculty of Science, University of Yaoundé I, P.O. Box 812, Yaoundé, Cameroon

**Keywords:** *Trichilia welw*i*tschii*, Cytotoxicity, Acetylcholinesterase, Nitric oxide, Limonoids

## Abstract

**Background:**

Limonoids are highly oxygenated compounds with a prototypical structure. Their occurrence in the plant kingdom is mainly confined to plant families of Meliaceae and Rutaceae. Owing to their wide range of pharmacological and therapeutic properties, this study was aimed at investigating the potential nitric oxide (NO) and acetylcholinesterase (AChE) inhibitory activity and the cytotoxicity of three limonoids: trichilia lactone D5 (**1**), rohituka 3 (**2**) and dregeanin DM4 (**3**), isolated from *Trichilia welwitschii* C.DC.

**Results:**

Results indicated that the three limonoids had low cytotoxicity towards Vero cells with LC_50_ values ranging from 89.17 to 75.82 μg/mL. Compounds (**2**) and (**3**) had lower cytotoxicity compared to puromycin and doxorubicin used as reference cytotoxic compounds. Compound (**1**) (LC_50_ of 23.55 μg/mL) had good antiproliferative activity against RAW 264.7 cancer cells. At the lowest concentration tested (0.5 µg/mL), compound (**2**) and (**3**) released the lowest amount of nitric oxide (2.97 and 2.93 µM, respectively). The three limonoids had anti-AChE activity with IC_50_ values ranged of 19.13 μg/mL for (**1**), 34.15 μg/mL for (**2**) and 45.66 μg/mL for (**3**), compared to galantamine (IC_50_ of 8.22 μg/mL) used as positive control.

**Conclusion:**

The limonoid compounds studied in this work inhibited nitric oxide production in LPS-stimulated macrophages and had anti-AChE activity. Trichilia lactone D5 had potential antiproliferative activity against RAW 264.7 cancer cells. The limonoids had low cytotoxicity towards Vero cells lines. This study provided further examples of the importance of limonoids compounds as potential AChE inhibitors and anti-inflammatory agents targeting the inhibition of NO production.

## Background

Nitric oxide (NO) is an important pro-inflammatory mediator involved in a wide variety of physiological and pathophysiological events; however overproduction of NO by inducible nitric oxide synthase (iNOS) results in severe inflammation [1]. An association between the development of cancer and inflammation has long-been documented [2]. Moreover, inflammation accelerates the appearance of some neurodegenerative disorders, such as Parkinson and Alzheimer’s diseases [3]. These insights are fostering new anti-inflammatory therapeutic approaches to cancer and neurodegenerative diseases development. There is no doubt that natural products remain important sources of new pharmaceutical compounds. Therefore, natural products research continues to explore a variety of compounds which may be used for the development of new drugs.

Limonoids are highly oxygenated compound with a prototypical structure, either containing or derived from a precursor with a 4,4,8-trimethyl-17-furanylsteroid skeleton. The prototypical structure consists of four six-membered rings and a furan ring. It is an important group of metabolically altered triterpenes, which are limited in their distribution. Their occurrence in the plant kingdom is mainly confined to plant families of Meliaceae and Rutaceae, and occurs less frequently in Cneoraceae and *Harrisonia *sp. of Simaroubaceae [4]. Limonoids isolated from the plant family Meliaceae are more complex with very high degree of oxidation and structural rearrangements [5]. In recent years a large number of pharmacological studies have been carried out to indicate their beneficial effects. Medicinal properties of limonoids reported include antibacterial, antifungal, antimalarial, anticancer and antiviral activities [6, 7]. In the last years, more than 100 limonoids have been isolated and characterized [8]. In our previous study, we reported the isolation and characterization of three limonoids compounds (dregeanin DM4, rohituka 3 and trichilia lactone D5) from *Trichilia welwitschii* C.DC. [9].

*Trichilia welwitschii* is a West African member of the Meliaceae growing as a large tree in the Terra Firma Forests of Nigeria, Cameroon, Angola and Gabon [10]. Species from the Meliaceae family and especially *Trichilia* genus have been well-documented for their ability to metabolize structurally diverse and biologically significant triterpenoids and limonoids [11]. No previous pharmacological study has been reported on compounds isolated from *T. welwitschii*. In our continue search of bioactive compounds from plants and owing to the wide range of pharmacological and therapeutic properties of limonoids, this study was carried out to investigate the potential antiproliferative, nitric oxide and acetylcholinesterase inhibitory activity of three limonoids isolated from *T. welwitschii*.

## Results and discussion

### Cytotoxicity study

Our goal was to determine whether the limonoid compounds exerted an inhibitory effect on cancer cell proliferation and their safety toward normal Vero cells. Therefore cytotoxic effect was evaluated against THP-1 and RAW 264.7 cancer cell lines and Vero cells as control. Doxorubicin and puromycin were used as standard anticancer drug. Puromycin is an aminonucleoside antibiotic, derived from the *Streptomyces alboniger* bacterium and used in cell biology as selective agent in cell culture systems for its toxicity to prokaryotic and eukaryotic cells [12]. Doxorubicin is commonly used to treat some leukemias and Hodgkin’s lymphoma, as well as cancers of the bladder, breast, stomach, lung, ovaries, thyroid, soft tissue sarcoma, multiple myeloma, and others [13]. The respective LC_50_ values and selectivity index (SI) are presented in Table 1. All the compounds had some level of toxicity which could be considered as weak or moderate cytotoxicity compared to puromycin and doxorubicin (LC_50_ values of 1.15–5.32 and 1.06–9.35 μg/mL respectively) used as reference cytotoxic compounds. The selectivity index (SI) values varied from 0.83 to 2.99 for limonoid compounds. The compounds were not toxic to Vero cells with LC_50_ values ranging from 89.17 to 75.82 μg/mL. The LC_50_ values varied between 81.20 and 84.53 μg/mL on THP-1. No significant antiproliferative activity was noted against the cancer cells with the exception of compound (**1**) with LC_50_ value of 23.55 μg/mL on RAW 264.7 cells. These results suggested that compounds **2** and **3** are not useful as antiproliferative therapeutic agent, due to their high IC_50_ concentration against THP-1 and RAW 264.7 cells. However, the potential antiproliferative effects of trichilia lactone D5 (**1**) against RAW 264.7 cancer cells is in line with previous reports on the antiproliferative activity of limonoids compounds [14–16].

**Table 1:** Cytotoxicity (LC_50_ in µg/mL**)** and the selectivity index (SI) of three limonoids isolated from *Trichilia welwitschii* and reference compounds (doxorubicin and puromycin) against cancer cell lines

Compounds	Vero	THP-1		RAW 264.7	
		LC_50_	SI	LC_50_	SI
**1**	89.17 ± 5.00^a^	81.20 ± 6.38^a^	0.87	23.55 ± 5.77^a^	2.99
**2**	85.22 ± 6.31^a^	81.20 ± 4.04^a^	0.87	65.68 ± 3.64^b^	1.07
**3**	75.82 ± 1.85^b^	84.53 ± 5.81^a^	0.83	61.86 ± 4.14^b^	1.14
Doxorubicin	9.35 ± 0.66^c^	nd	nd	1.06 ± 0.65^c^	66.42
Puromycin	5.32 ± 0.90^d^	0.4 ± 0.02^b^	176.03	1.15 ± 0.17^c^	61.23

Values with different letters are significantly different at p < 0.05

*nd* not determined

### NO inhibitory activity

**Fig. 1:**
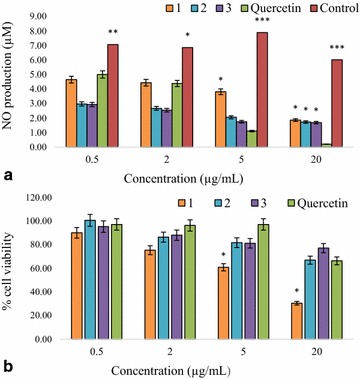
Inhibitory activity of three limonoids isolated from *Trichilia welwitschii* on nitrix oxide production. The RAW 264.7 macrophages cells were seeded in 96 well-microtitre plates and were activated by incubation in medium containing 1 µg/mL LPS alone (control) or lipopolysaccharide with different concentrations of the samples dissolved in DMSO. Nitric oxide released from macrophages was determined by measuring the nitrite concentration in culture supernatant using the Griess reagent. The concentrations of nitrite were derived from regression analysis using serial dilutions of sodium nitrite as a standard. Percentage inhibition was calculated based on the ability of compounds to inhibit nitric oxide formation by cells compared with the control (cells in media without compounds), which was considered as 0 % inhibition. Data represent the mean ± SE of three independent experiments ∗p < 0.05, ∗p < 0.01 and ∗∗∗p < 0.001 are significantly different from the reference compound quercetin

Macrophages produce inflammatory mediators including NO in response to bacterial LPS; NO plays a pivotal role in many body functions; however, its overproduction can lead to cytotoxicity and inflammation [17]. Therefore, NO inhibitors are essential for preventing inflammatory diseases. Nitric oxide plays an important role in the inflammatory process, and an inhibitor of NO production and may be considered as a potential anti-inflammatory agent. Therefore, NO inhibitors are essential for preventing inflammatory diseases. Quercetin has been reported to significantly suppress NO production in LPS-stimulated RAW 264.7 murine macrophage cell line [18]. In this study, RAW 264.7 macrophages were treated with LPS and various concentrations of limonoid compounds and quercetin as control, then NO production and cell viability were measured. The limonoid compounds had a concentration dependent inhibition on NO production induced by lipopolysaccharide (LPS) in macrophages (Fig. 1a). At the lowest concentration (0.5 µg/mL), compound (**2**) and (**3**) released the lowest amount nitric oxide (2.97 and 2.93 µM, respectively). The cytotoxicity of compounds against RAW 264.7 macrophages was also tested by MTT assay (Fig. 1b). Compound (**1**) had slight toxic effect; while the two other compounds did not had significant cytotoxicity at the concentration leading to effective inhibition of NO production. For the two other limonoids compounds, 2-hydroxyxylorumphiin F and xylorumphiin I have been described a moderate inhibitory activity against nitric oxide production from LPS-activated macrophages with IC_50_ values of 24.5 and 31.3 μM, respectively [19]. Additionally, for six limonoids including trichilinin B (1), 4, ohchinin (7), 23-hydroxyohchininolide (8), 21-hydroxyisoohchininolide (9), 10, and methyl indole 3-carboxylate (12), have been described and inhibited production of NO with IC_50_ values in the range of 4.6–87.3 μM and with no toxicity to the cells [14]. Our results provided further examples of the importance of limonoid compounds as potential anti-inflammatory agents targeting NO inhibition.

### Acetylcholinesterase inhibition

Recognized factors in Alzheimer’s disease include free radicals, inflammation of the brain tissue and acetylcholine deficiency. Therefore, acetylcholine breakdown in the brain can be prevented by the inhibition of acetyl cholinesterase activity, leading to an increase in acetylcholine concentration. In the present study, limonoid compounds were investigated for their ability to inhibit the activity of AChE comparatively to galantamine. Results are presented in Table 2. Galantamine is a reversible cholinesterase inhibitor used for the treatment of mild to moderate Alzheimer’s disease and various other memory impairments [20]. Compare to galantamine (IC_50_ of 8.22 μg/mL) used as standard AChE inhibitor; the three limonoids isolated from *Trichilia welwitschii* had weak to moderate inhibitory activities. At 50 µg/mL, the inhibitory potential of limonoids **1**, **2** and **3** was 94.33, 82.67 and 87 % respectively. At the lowest concentration (6.25 µg/mL), the AChE inhibitory activity of the three compounds dropped to 28.00, 14.00 and 30.33 % for limonoids 1, 2 and 3 respectively. Concentration dependent inhibition was also observed and the IC_50_ values ranged from 19.13 μg/mL for compound (**1**) to 34.15 μg/mL for compound (**2**) and 45.69 μg/mL for compound (**3**). However, all the samples had lower inhibitory activities compare with that of galantamine (IC_50_ 8.22 μg/mL). Not much work has been done on the AChE activity of *Trichilia* species [21]. There is a widespread effort to find new AChE inhibitors from natural source, but not much work has focused on limonoid compounds. Our compounds had a much higher anti- AChE activity than the limonoids from *Kigelia africana* (Bignoniaceae) with IC_50_ values in the ranges 137.5–225.2 μM [22].

**Table 2:** Acetylcholinesterase inhibitory activity of three limonoids isolated from *Trichilia welwitschii* and reference compound (galantamine)

Compounds	Concentration (µg/mL)	% AChE inhibition	IC_50_ (µg/mL)
**1**	50	94.33 ± 8.15	19.13 ± 0.41^a^
	25	71.67 ± 6.03	
	12.5	18.00 ± 8.89	
	6.25	28.00 ± 6.56	
**2**	50	82.67 ± 7.04	34.15 ± 1.66^b^
	25	67.00 ± 4.36	
	12.5	47.33 ± 2.08	
	6.25	14.00 ± 6.00	
**3**	50	87.00 ± 8.74	45.69 ± 3.65^c^
	25	70.67 ± 3.50	
	12.5	31.67 ± 5.20	
	6.25	30.33 ± 3.51	
Galantamine	20	83.00 ± 8.89	8.22 ± 2.73^d^
	5	69.67 ± 4.16	
	2	57.67 ± 4.04	
	0.5	38.00 ± 1.00	

Values with different letters are significantly different at p < 0.05

## Conclusion

The limonoid compounds studied in this work inhibited nitric oxide production in LPS-stimulated macrophages and presented AChE inhibitory activity. They had low cytotoxicity against Vero cells lines. The potential antiproliferative effect of compound against RAW 264.7 cancer cells was also demonstrated. This study provided further examples of the importance of limonoid compounds as potential AChE inhibitors and anti-inflammatory agents targeting the inhibition of NO production.

## Methods

### Chemicals

Sodium dodecyl sulphate, bovine serum albumin (BSA), sodium chloride (NaCl), MgCl_2_·6H_2_O, acetylthiocholine iodide (ATCI), galantamine, 5,5-dithiobis-2-nitrobenzoic acid (DTNB), acetylcholinesterase (AChE) enzyme from electric eels (type VI-S lyophilized powder), sodium nitrite, ferrous sulfate, indomethacin and 15-lipoxygenase from *Glycine max* purchased from Sigma (Germany) and Tris(hydroxymethyl)aminomethane from Sigma, (Switzerland). Foetal calf serum (FCS), penicillin/streptomycin/fungizone (PSF) and Dulbecco’s modified Eagle’s medium (DMEM) were obtained from Highveld Biological Products (South Africa). Phosphate buffered saline (PBS) and trypsin were purchased from Whitehead Scientific (South Africa). Quercetin, 3-(4,5-dimethylthiazol-2-yl)-2,5-diphenyl-tetrazolium bromide (MTT) were purchased from Sigma-Aldrich St. Louis, MO, USA.

### Limonoids compounds

**Fig. 2:**
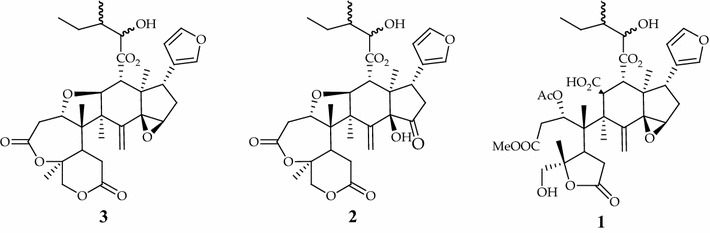
Compounds isolated from *T. welwitschii*. **1** Trichilia lactone D5; **2** rohituka 3; **3** dregeanin. The isolation procedure and the structure elucidation of the compounds were previously described [9]

The three limonoids compounds (dregeanin DM4, rohituka 3 and trichilia lactone D5) studied in this work were isolated from seeds of *Trichilia welwitschii.* We previously described the isolation procedure and the structure elucidation of the compounds [9]. Chemical structures are shown in Fig. 2.

### Cytotoxicity

#### Cell culture

Cells lines including human monocytic THP-1, murine macrophage RAW 264.7 and the Vero monkey kidney cell lines were obtained from the American Type Culture Collection (Rockville, MD, USA). They were maintained in DMEM supplemented with 10 % fetal calf serum (FCS) and 1 % penicillin/streptomycin/fongizone (PSF) under standard cell culture conditions at 37 °C and 5 % CO_2_ in a humidified environment.

#### MTT assay

The cytotoxicity of compounds was determined by the 3-[4,5-dimethylthiazol-2-yl]-2,5-diphenyltetrazolium (MTT) assay as previously described [23]. The selectivity index (SI) values were calculated by dividing cytotoxicity LC_50_ values of normal Vero cells by the LC_50_ of cancer cells in the same units.

### Nitric oxide inhibitory activity and viability of LPS-activated RAW 264.7 macrophages

The RAW 264.7 macrophages cells were seeded in 96 well-microtitre plates and were activated by incubation in medium containing 1 µg/mL LPS alone (control) or lipopolysaccharide with different concentrations of the samples dissolved in DMSO. Quercetin served as a positive control NO inhibitor for the reduction of NO production [18].

#### Measurement of nitrite

Nitric oxide released from macrophages was determined by measuring the nitrite concentration in culture supernatant using the Griess reagent. After 24 h incubation, 100 µL of supernatant from each well of cell culture plates was transferred into 96-well microtitre plates and an equal volume of Griess reagent was added. The absorbance of the resultant solutions was determined on a BioTek Synergy microplate reader after 10 min at 550 nm. The concentrations of nitrite were derived from regression analysis using serial dilutions of sodium nitrite as a standard. Percentage inhibition was calculated based on the ability of compounds to inhibit nitric oxide formation by cells compared with the control (cells in media without compounds), which was considered as 0 % inhibition.

#### Cell viability

To determine whether the observed nitric oxide inhibition was not due to cytotoxic effects, MTT assay was also performed on the macrophage cells as previously described [23].

### Acetylcholinesterase inhibition activity

Inhibition of acetylcholinesterase activity was determined using Ellman’s colorimetric method [24] with slight modifications. In a 96-well plate was placed: 25 µL of 15 mmol/L ATCI in water, 125 µL of 3 mmol/L DTNB in Buffer A (50 mmol/L Tris–HCl, pH 8.0, containing 0.1 mol/L NaCl and 0.02 mol/L MgCl_2_·6H_2_O), 50 µL of Buffer B (50 mmol/L, pH 8, containing 0.1 % bovine serum albumin) and 25 µL of samples (serially diluted: 500–3.9 µg/mL for extract and fraction and 100–0.78 µg/mL for compounds and galantamine used as AChE standard inhibitor). Then, AChE (0.2 U/mL) was added to the wells and the absorbance was determined spectrophotometrically (BioTek Synergy microplate reader) at 405 nm. Distilled water was used as negative control. The percentage of inhibition was calculated as follow:

$$\begin{aligned}{\text{V}} &= \Delta_{\text{Abs}} /\Delta_{\text{t}} , \\ \% {\text{enzyme activity}} &= \left( {{\text{V}}/{\text{V}}_{ \text{max} } } \right) \times 100, \\ \% {\text{enzyme inhibition}} &= 100 - \% {\text{enzyme activity}}\end{aligned}$$

where V is the rate of the reaction in the presence of inhibitor and V_max_ is the reaction’s rate of the control without inhibitor. The IC_50_ values of samples leading to 50 % inhibition were calculated by plotting the percentage of inhibition against the concentrations.

## Statistical analysis

All results are presented as means of triplicate experiments. All experiments were conducted in triplicate and values expressed as mean ± standard deviation. Statistical analysis was performed with GraphPad InStat Software and results were compared using the Student-Newman Keul test at 5, 1 or 0.1 % significance level.

## Abbreviations

NO: nitric oxide
AChE: acetylcholinesterase
ATCI: acetylthiocholine iodide
DTNB: 5,5-dithiobis-2-nitrobenzoic acid
DMEM: Dulbecco’s modified Eagle’s medium
